# Brain Magnetic Resonance Imaging Reveals Different Courses of Disease in Pediatric and Adult Cerebral Malaria

**DOI:** 10.1093/cid/ciaa1647

**Published:** 2020-12-16

**Authors:** Praveen K Sahu, Angelika Hoffmann, Megharay Majhi, Rajyabardhan Pattnaik, Catriona Patterson, Kishore C Mahanta, Akshaya K Mohanty, Rashmi R Mohanty, Sonia Joshi, Anita Mohanty, Jabamani Bage, Sameer Maharana, Angelika Seitz, Martin Bendszus, Steven A Sullivan, Ian W Turnbull, Arjen M Dondorp, Himanshu Gupta, Lukas Pirpamer, Sanjib Mohanty, Samuel C Wassmer

**Affiliations:** 1 Center for the Study of Complex Malaria in India, Ispat General Hospital, Rourkela, Odisha, India; 2 Department of Neuroradiology, University Hospital Heidelberg, Heidelberg, Germany; 3 University Institute of Diagnostic and Interventional Neuroradiology, University Hospital Bern, Inselspital, University of Bern, Switzerland; 4 Department of Radiology, Ispat General Hospital, Rourkela, Odisha, India; 5 Department of Intensive Care, Ispat General Hospital, Rourkela, Odisha, India; 6 Department of Infection Biology, London School of Hygiene and Tropical Medicine, London, United Kingdom; 7 Infectious Diseases Biology Unit, Institute of Life Sciences, Bhubaneswar, Odisha, India; 8 Department of Ophthalmology, Ispat General Hospital, Rourkela, Odisha, India; 9 Department of Biology, New York University, New York, New York, USA; 10 North Manchester General Hospital, Manchester, United Kingdom; 11 Mahidol Oxford Tropical Medicine Research Unit, Faculty of Tropical Medicine, Mahidol University, Bangkok, Thailand; 12 Centre for Tropical Medicine and Global Health, Nuffield Department of Clinical Medicine, Oxford, United Kingdom; 13 Department of Neurology, Division of Neurogeriatrics, Medical University of Graz, Graz, Austria

**Keywords:** cerebral malaria, *Plasmodium falciparum*, magnetic resonance imaging, apparent diffusion coefficient maps, hypoxia

## Abstract

**Background:**

Cerebral malaria is a common presentation of severe *Plasmodium falciparum* infection and remains an important cause of death in the tropics. Key aspects of its pathogenesis are still incompletely understood, but severe brain swelling identified by magnetic resonance imaging (MRI) was associated with a fatal outcome in African children. In contrast, neuroimaging investigations failed to identify cerebral features associated with fatality in Asian adults.

**Methods:**

Quantitative MRI with brain volume assessment and apparent diffusion coefficient (ADC) histogram analyses were performed for the first time in 65 patients with cerebral malaria to compare disease signatures between children and adults from the same cohort, as well as between fatal and nonfatal cases.

**Results:**

We found an age-dependent decrease in brain swelling during acute cerebral malaria, and brain volumes did not differ between fatal and nonfatal cases across both age groups. In nonfatal disease, reversible, hypoxia-induced cytotoxic edema occurred predominantly in the white matter in children, and in the basal ganglia in adults. In fatal cases, quantitative ADC histogram analyses also demonstrated different end-stage patterns between adults and children: Severe hypoxia, evidenced by global ADC decrease and elevated plasma levels of lipocalin-2 and microRNA-150, was associated with a fatal outcome in adults. In fatal pediatric disease, our results corroborate an increase in brain volume, leading to augmented cerebral pressure, brainstem herniation, and death.

**Conclusions:**

Our findings suggest distinct pathogenic patterns in pediatric and adult cerebral malaria with a stronger cytotoxic component in adults, supporting the development of age-specific adjunct therapies.

## Teaser

Using quantitative magnetic resonance imaging analyses of brain volume and cerebral tissue water diffusion, we demonstrate that children and adults with cerebral malaria have a different disease evolution and show that pronounced, global brain hypoxia is associated with death in adults.

Falciparum malaria remains the most important parasitic disease globally. In high-transmission settings in sub-Saharan Africa, falciparum malaria is a pediatric disease; in lower-transmission settings such as Southeast Asia, all age groups are affected, owing to differences in antimalaria immunity building [1]. Cerebral malaria (CM) is the presenting syndrome in around half of the patients with severe malaria, both in children and adults. CM leads to neurological dysfunctions including seizures and impaired consciousness and has a fatality rate up to 30% in treated patients [2]. The range and type of CM-associated complications vary between the 2 age groups: While children more frequently develop cerebral involvement as mono-organ failure, adults often present with additional organ dysfunctions such as acute kidney injury, jaundice, and acute respiratory distress syndrome. Such differences in clinical presentation were reported between cohorts from different geographic areas [3], as well as within the same cohort [4].

The fundamental pathogenesis of fatal CM is still incompletely understood. The mechanical obstruction of cerebral microvessels by sequestered *Plasmodium falciparum*–parasitized red blood cells (pRBCs) is central to its pathogenesis [5], and hyperactivation of host immune cells leading to the excessive release of proinflammatory cytokines, as well as critical hematologic dysfunctions, has also been proposed [6].

The causes and the contribution of brain swelling to neurological symptoms have been a source of debate [7]. Brain swelling occurs frequently in both adult [8, 9] and pediatric CM [10]. In African children, the increase in cerebral volume can be severe and result in brain stem herniation, leading to death by respiratory arrest [11]. This differs markedly from Southeast Asian adults, who usually present milder cerebral swelling not leading to coma or death [12].

Specific magnetic resonance imaging (MRI) techniques may help to get further insights into disease pathology. Apparent diffusion coefficient (ADC) is a measure of the magnitude of diffusion of water molecules within a tissue. ADC maps are commonly calculated clinically using MRI with diffusion-weighted imaging and allow the discrimination between cytotoxic and vasogenic edema. Cytotoxic edema is characterized by ADC decrease due to restricted diffusion of water molecules [13]: ATP pumps cease to operate following a hypoxic/hypoglycemic injury, leading to a shift of fluid from the extracellular to the intracellular compartment and shrinkage of the extracellular space [14]. Conversely, vasogenic edema is characterized by an increase in ADC and expansion of the extracellular space after leakage of fluid from the blood-brain barrier to the parenchymal tissue. To date, no systematic quantitative ADC studies have been performed in CM patients to distinguish between these different etiologies of brain swelling.

We provide the first comprehensive comparative analysis of CM-associated structural and functional brain changes in a cohort of both pediatric and adult Indian patients, and investigate patterns associated with survival and mortality by combining MRI with quantitative brain volume and ADC histogram analyses, complemented with an assessment of parasite biomass and hypoxia biomarkers.

## PATIENTS AND METHODS

### Study Site and Patients

The study was carried out at Ispat General Hospital (IGH) in Rourkela, India, from October 2013 to November 2019 (Supplementary Table 1). Written consent was obtained from all enrolled subjects or their families prior to inclusion in the study. Ethical approval was obtained from IGH, the Indian Council of Medical Research (TDR589/2010/ECDII), New York University School of Medicine (S12-03016), the London School of Hygiene and Tropical Medicine, and Heidelberg University Hospital. Eighty-five patients were enrolled, and MRI was carried out within 10 hours of admission. Sixty-five patients (76%) underwent MRI a second time (Table 1; Supplementary Figure 1). Patients were classified as follows:

**Table 1.** Summary of Clinical and Imaging Findings in 91 Indian Patients with CM or UM

		Children		Adults		
Demographics		Fatal CM	Non-fatal CM	Fatal CM	Non-fatal CM	UM
	No.	3	24	4	34	20
	Age (mean, (SD))	5 (0)	7.8 (3.9)	38 (9.3)	31 (9.1)	36 (13)
	Sex (female, male)	0, 3	6, 18	0, 4	10, 22	6, 14
**Parasite burden**						
	Parasitemia: No.	3	22	4	32	19
	Parasitemia (×1000/uL): median (range)	0.43 (0.17 71.36)	4.33 (0.41 20.78)	97.50 (1.48 294.02)	4.08 (0.39 22.59)	28.48 (0.60 192.01)
	PfHRP2: No.	3	19	3	20	9
	PfHRP2 (ng/mL): median (range)	13.71 (9.05 1019.72)	933.50 (140.13 1186.64)	10.34 (9.10 606.95)	933.77 (133.73 1293.01)	275.57 (9.06 640.75)
**Clinical parameters**						
	No.	3	24	4	34	20
	Platelet count (×1000/uL): median (range)	47.00 (31.25 48.50)	47.50 (27.00 81.50)	8.00 (0.00 40.00)	36.25 (15.00 60.00)	58.50 (38.00 85.50)
	Hb (g/dL): median (range)	5.60 (4.03 7.25)	6.65 (6.00 7.85)	9.00 (7.50 10.25)	7.40 (6.20 9.80)	10.75 (9.40 12.20)
	Bilirubin (mg/dL): median (range)	1.40 (0.65 2.75)	1.95 (0.65 2.90)	26.90 (23.65 29.60)	3.60 (2.10 7.50)	1.30 (0.95 1.75)
	Creatinine (mg/dL): median (range)	1.70 (0.95 4.48)	0.60 (0.50 0.90)	6.75 (5.05 8.10)	2.35 (1.20 5.00)	1.00 (0.80 1.10)
	Retinopathies: hemmorhages (1/0): No. (%)	3 (33%)	23 (57%)	4 (50%)	30 (50%)	18 (11%)
	Retinopathies: whitening (1/0): No. (%)	3 (0%)	23 (9%)	4 (0%)	30 (17%)	18 (0%)
	Retinopathies: papilledema: (1/0): No. (%)	3 (0%)	23 (4%)	4 (0%)	30 (13%)	18 (0%)
**Quantitative MRI parameters**						
** Brain volume**						
First scan	No.	2	15	4	23	18
	Normalized brain volume (cm3): mean (SD)	2070.73 (5.04)	1684.94 (695.98)	1594.94 (148.09)	1639.53 (118.35)	1572.64 (109.13)
Second scan	No.	NA	48.11 (44.22 74.98)	NA	47.92 (46.89 63.25)	50.37 (48.23 71.00)
	Time between first and second scan (hours): median (IQR)	NA	1627.95 (701.05)	NA	1665.14 (148.47)	1554.95 (80.97)
	Normalised brain volume (cm3): mean (SD)					
** ADC**		3	16	3	28	17
First scan	No.	781.50 (637.50 797.62)	782.00 (759.75 798.75)	598.50 (579.00 630.00)	677.50 (627.50 709.75)	742.50 (732.88 760.00)
	Whole brain adc_peak_ (10^˗6^ mm^2^/s): median (range)	0	14	0	19	14
Second scan	No.	NA	50.28 (47.18 96.03)	NA	48.02 (47.42 66.75)	50.33 (48.10 70.88)
	Time between first & second scan (hours): median (range)	NA	778.50 (757.50 800.00)	NA	718.50 (672.75 734.50)	716.25 (700.50 739.50)
	Whole brain ADC_peak_ (10^˗6^ mm^2^/s): median (range)	NA	48.11 (44.22 74.98)	NA	47.92 (46.89 63.25)	50.37 (48.23 71.00)

Abbreviations: 1/0, present/absent; NA, Not available (not performed); No., number of samples; SD, standard deviation; ADC, apparent diffusion coefficient.

• CM (n = 65): All CM patients fulfilled the strict clinical definition, according to the World Health Organization criteria [2], including a Glasgow Coma Score ≤9/15 for adults and a Blantyre Coma Score ≤2 for young preverbal children. Inclusion and exclusion criteria are detailed in the Supplementary Materials. Two patients were first diagnosed with uncomplicated malaria (UM), but developed CM after antimalarial treatment. In this cohort, the case fatality rate in patients with CM was 7 of 65 (10.8%).
• UM (n = 26): fully conscious UM patients (Glasgow Coma Score = 15/15 for adults) infected with *P. falciparum* with fever (axillary temperature, ≥37.5°C) or history of fever in the preceding 24 hours, and no signs of complicated malaria, were eligible for inclusion. Because of the difficulty to obtain quality MRI scans in young children with febrile illness, pediatric UM control patients were not included in this study.

### Study Procedures and Clinical Care

On admission, a full medical history and physical examination including funduscopy were conducted and recorded on a standardized clinical record form. Blood samples were collected for complete blood count, parasite count, hemoglobin, hematocrit, glucose, and biochemistry. Antimalarial treatments were in accordance with the national drug policy of the government of India. Additional details on funduscopy and drug regimens are available in the Supplementary Materials.

### MRI and Analysis

Imaging of the brain was performed using a 1.5T Siemens Symphony MRI scanner (Siemens AG, Erlangen, Germany). ADC maps were generated and used for differentiation between cytotoxic and vasogenic edema [15]. Each MRI was interpreted by 1 radiologist on site (M. M.) and 2 neuroradiologists off-site (I. W. T. and A. H.). T1-weighted images were used for automated volume analysis using the freely available program SIENAX to assess brain volume, and SIENA to compare admission and follow-up scans (Supplementary Figures 2 and 3) [16]. Assessments were performed blindly by an experienced MRI postprocessing image analysis expert (L. P.). Normalized ADC histograms were created for whole brain, and the peak location of whole-brain histograms, corresponding to the most common ADC value in the brain tissue [17], was used for the analyses. Additional details are available in the Supplementary Materials.

### Plasma Level Evaluation of Lipocalin-2, MicroRNA-150, and *P. falciparum* Histidine-Rich Protein 2

Plasma levels of lipocalin-2, a recently described marker of brain hypoxia [18], microRNA-150 (miRNA-150), a regulator of hypoxia-induced factor 1α [19], and *P. falciparum* histidine-rich protein 2 (PfHRP2), an indicator of the total parasite biomass [20], were assessed in all patients from the cohort using commercially available kits (Supplementary Materials). All assays were performed according to the manufacturer protocols, in duplicate with results averaged for analyses, and by individuals blinded to study endpoints.

### Statistical Analyses

The χ ^2^ test was used to compare categorical variables. Depending on the normality distribution, an unpaired Student *t* test or Mann-Whitney test was used to compare 2 groups. Pearson correlation coefficients were calculated for correlation analyses. A 2-sided *P* < .05 was considered statistically significant. All statistical analyses were performed using GraphPad Prism 8.3 (GraphPad Software).

### Data Availability

Anonymized evaluation data are available upon request.

## RESULTS

### Brain Volume Increase in CM Is Age-Dependent and Not Associated With Mortality

**Figure 1.**
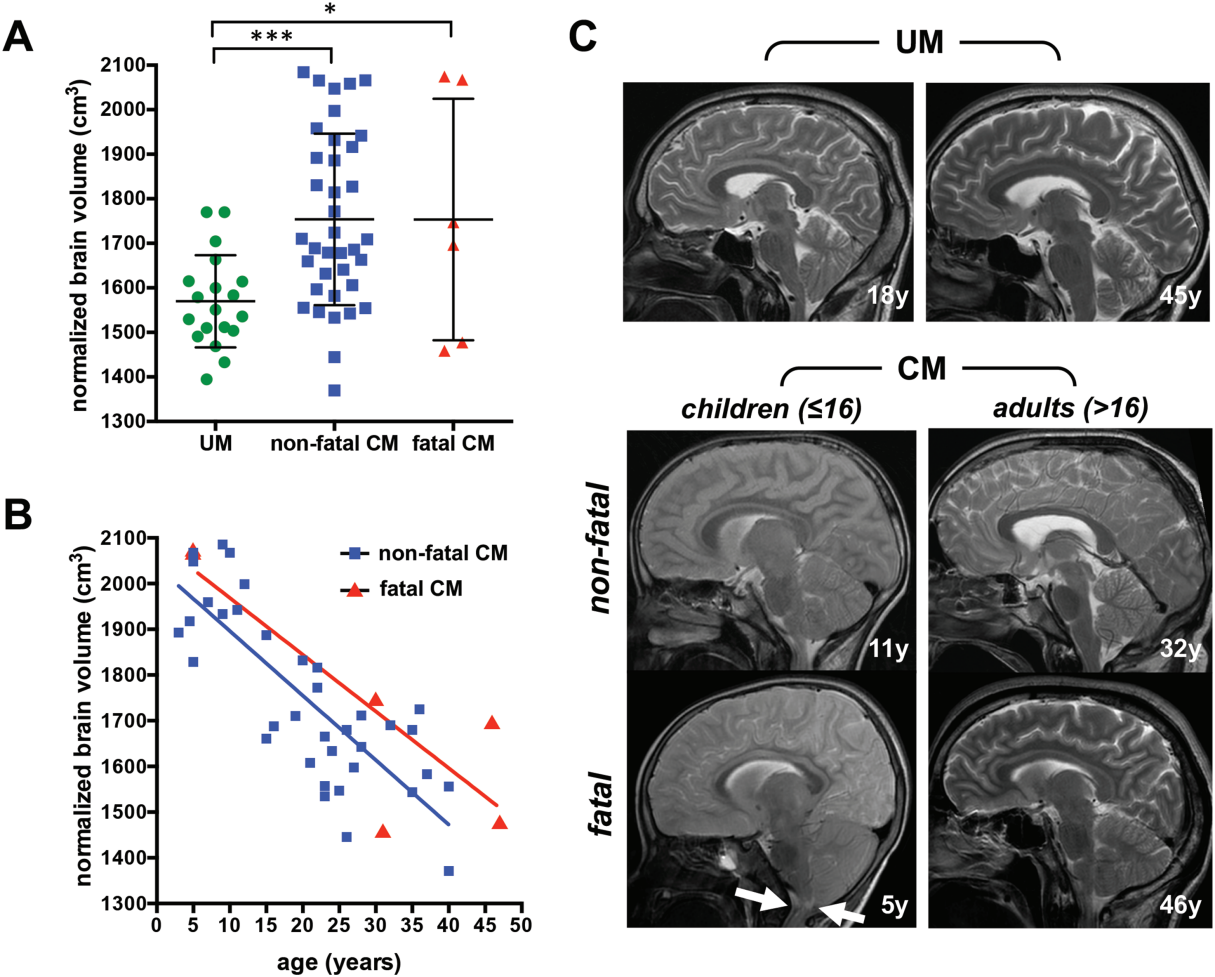
Comparison of brain volumes on admission between age and disease groups. *A*, Normalized brain volumes in uncomplicated malaria (UM) and nonfatal and fatal cerebral malaria (CM). *B*, Correlation between age and normalized brain volume in nonfatal and fatal CM. *C*, Representative sagittal T2-weighted magnetic resonance imaging of patients with UM (first row) and CM (second and third rows). In pediatric CM patients, the outer cerebrospinal fluid spaces are more reduced due to brain swelling compared with adults. One fatal pediatric CM case showed brain stem herniation with no remaining cerebrospinal fluid space at the craniocervical junction (arrows). *P < .05; ***P < .0005..

Normalized brain volume on admission was higher in patients with nonfatal CM (1753.7 ± 192.5 cm^3^, *P* = .0003) and fatal CM (1753.5 ± 271.2 cm^3^, *P* = .019) compared with UM patients (1570 ± 103 cm^3^), irrespective of age. There was no significant difference in volume between fatal and nonfatal CM, and large intragroup variations were observed, ranging from 1396.6 to 2084.4 cm^3^ in nonfatal CM, and from 1458.9 to 2074.3 cm^3^ in fatal CM (Figure 1A). The normalized brain volume on admission was negatively correlated with age, irrespective of the outcome (Figure 1B; *R*^2 ^= 0.66, *P* < .0001 for nonfatal CM; *R*^2 ^= 0.73, *P* = .029 for fatal CM). At the time of MRI, 1 of 3 fatal pediatric CM patients showed signs of brain stem herniation (Figure 1C). The other 2 patients developed end-stage disease 47 and 49 hours after the scan, respectively. Fatal adult CM patients showed moderate (2/4) or no brain swelling (2/4), and no signs of brain stem herniation were observed (Figure 1C). In nonfatal CM, reversible brain swelling after treatment was evidenced by a rapid decrease in brain volume at the follow-up MRI compared to the volume measured on admission (*P* = .014; Supplementary Figure 4*A* and 4*C*), which is consistent with the previously reported semi-quantitative assessment of 11 cases from the same cohort [8]. UM patients showed no significant change in brain volume between the 2 scans (Supplementary Figure 4*B* and 4*D*).

### Nonfatal Pediatric and Adult CM Patients Show Reversible Cytotoxic Edema in Different Brain Regions

**Figure 2.**
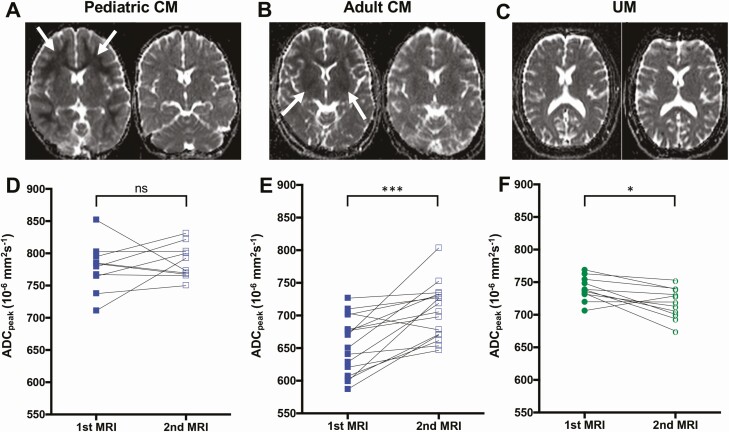
Evolution of apparent diffusion coefficient (ADC) between admission and follow-up magnetic resonance imaging (MRI). *A*, *C*, and *E*, Representative sequential ADC maps at the same window level (130) and width (290) from 3 cases of pediatric cerebral malaria (CM), adult CM, and uncomplicated malaria (UM). *A*, Pediatric CM patient showing decreased ADC values in the white matter on admission (left, arrows) that resolved at follow-up (right). *C*, Adult CM patient showing decreased ADC values in the basal ganglia on admission (left) that reversed at follow-up (right). *E*, UM patient without pathological ADC changes on admission (left) or at follow-up (right). Whole-brain ADC_peak_ values on admission and at follow-up are displayed and grouped according to age and disease categories: nonfatal pediatric CM (*B*), nonfatal adult CM (*D*), and UM (*F*). *P < .05; ***P < .0005; ns, not significant.

In nonfatal pediatric CM, the most prominent finding was diffusion restriction in the deep and subcortical white matter characterized by a decrease in ADC, indicating hypoxia-related cytotoxic edema (Figure 2A). In contrast, an ADC decrease in the basal ganglia was the hallmark of nonfatal adult CM, indicating focal hypoxia-related cytotoxic edema in deep gray matter structures (Figure 2B). UM patients did not present any pathological features noticeable by visual inspection (Figure 2E). In both age groups, serial ADC measures revealed rapid ADC reversal in follow-up scans, with only subtle remaining changes in a few patients (median of 47.9 hours [interquartile range {IQR}, 7.8 hours] after the first scan), indicative of reversible cytotoxic edema (Figure 2D and 2E; Supplementary Figure 5; Table 1). In pediatric CM, ADC_peak_ values increased at follow-up in 50% of pediatric CM cases (5/10). The low ADC_peak_ values in these children were mainly located in the cerebral white matter. Thirty percent of pediatric CM patients (3/10) with increased ADC_peak_ values on admission showed decreased values at follow-up, suggesting a predominantly vasogenic component during the acute disease. In 20% of cases (2/10), ADC_peak_ values remained constant (Figure 2A and 2D). In adult CM patients, ADC_peak_ values increased significantly at follow-up compared to admission (677.50 × 10^–6^mm^2^/second [IQR, 82.25] vs 718.50 × 10^–6^mm^2^/second [IQR, 61.75]; *P* = .0004; Figure 2E), reaching values similar to ADC_peak_ values observed in most UM patients (724.25 × 10^–6^mm^2^/second [IQR, 39.00]). ADC_peak_ values in UM patients slightly decreased at follow-up (*P* = .012), indicating a resolution of mild vasogenic edema posttreatment (Figure 2C and 2F; Table 1).

### Cytotoxic Edema Is Associated With Adult CM and Is More Severe in Fatal Disease

**Figure 3.**
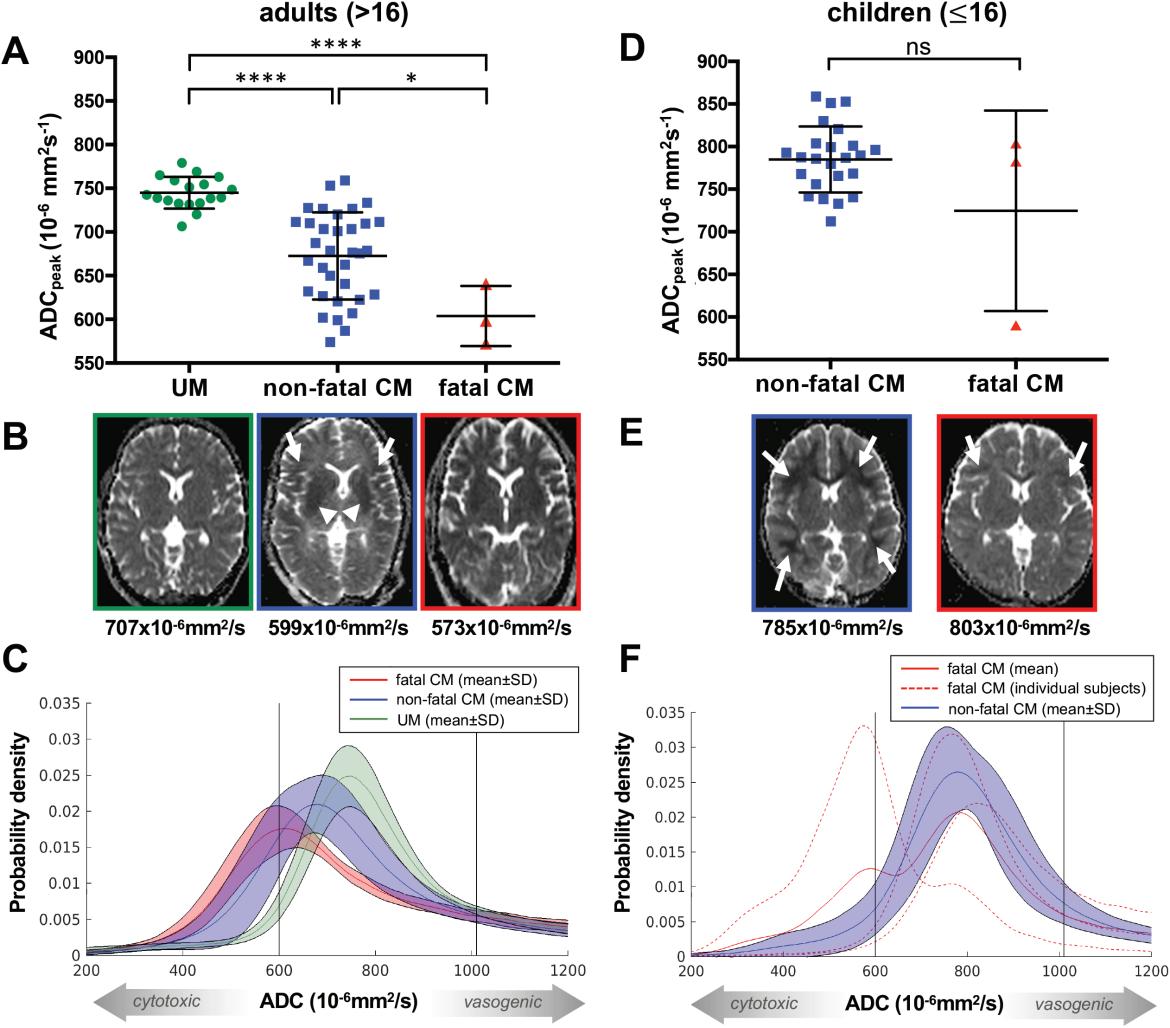
Apparent diffusion coefficient (ADC) alterations in adult and pediatric cerebral malaria (CM). *A*, Whole-brain ADC_peak_ values (mean and standard deviation) of adult patients with uncomplicated malaria (UM), nonfatal CM, and fatal CM. *B*, Representative ADC maps at the same window level (130) and width (290) from 1 representative UM patient (framed in green), 1 case of nonfatal adult CM (framed in blue), and 1 case of fatal adult CM (framed in red) are shown with the corresponding ADC_peak_ value listed below the image. The ADC map of the representative UM patient does not show pathological alterations, whereas the nonfatal case exhibits an ADC decrease in the basal ganglia (white arrowheads) and the subcortical white matter (white arrows). In the fatal CM case, a global ADC decrease, which is hardly detectable by visual inspection, results in low whole-brain ADC_peak_ values. *C*, Mean histograms with mean and standard deviation of UM, nonfatal adult CM, and fatal adult CM. ADC_peak_ values were the lowest in fatal CM, followed by nonfatal CM and UM. *D*, Whole-brain ADC_peak_ values (mean and standard deviation) of pediatric patients with nonfatal CM and fatal CM. *E*, Representative ADC images at the same window level (130) and width (290) from 2 nonfatal pediatric cases (framed in blue) and 2 fatal pediatric CM cases (framed in red) with the corresponding ADC_peak_ value listed below the image. The displayed ADC map of a nonfatal case illustrates a strong ADC decrease in the white matter (white arrows). The ADC map of 1 fatal pediatric case shows globally elevated ADC values with subtle ADC decrease in the subcortical white matter (white arrows). *F*, The mean histogram with mean and standard deviation of nonfatal pediatric CM and the mean histogram of fatal pediatric CM cases as well as the individual histograms of fatal pediatric CM cases. *P < .05; ****P < .0001; ns, not significant.

Adult patients with fatal CM had significantly lower ADC_peak_ values compared to patients from the same age group who survived (*P* = .026; Figure 3A; Table 1). UM had no pathological ADC alteration by visual inspection, whereas in adults with nonfatal CM, local areas of ADC decrease were observed in the subcortical white matter and the basal ganglia (Figure 3B). In contrast, patients with fatal disease showed a homogeneous, global ADC decrease affecting all brain structures (Figure 3B). Such homogeneous, symmetric diffusion alterations can only be detected by measurement of ADC values, and this was evidenced by a shift of ADC histograms to lower values (Figure 3C; Supplementary Figure 6). Pediatric CM patients showed slightly decreased, normal and high ADC_peak_ values (Figure 3D and 3E; Supplementary Figure 6; Table 1). Compared to adults, they presented a less pronounced cytotoxic component. ADC decrease affecting a large proportion of the white matter was seen in patients with slightly decreased ADC_peak_ values (Figure 3D). Two of 3 fatal CM cases had no or only little subcortical ADC decrease and showed normal to high ADC_peak_ values and thus no cytotoxic edema, but rather an increased water content, consistent with vasogenic or interstitial edema (Figure 3D and 3E; Supplementary Figure 6; Table 1). In the fatal case with brain stem herniation, ADC_peak_ values were significantly decreased, suggesting severe cytotoxic brain swelling caused by brain stem herniation as an end stage of fatal pediatric CM (Supplementary Figure 7).

### Plasma Biomarkers of Hypoxia Reflect Changes in ADC During CM

**Figure 4.**
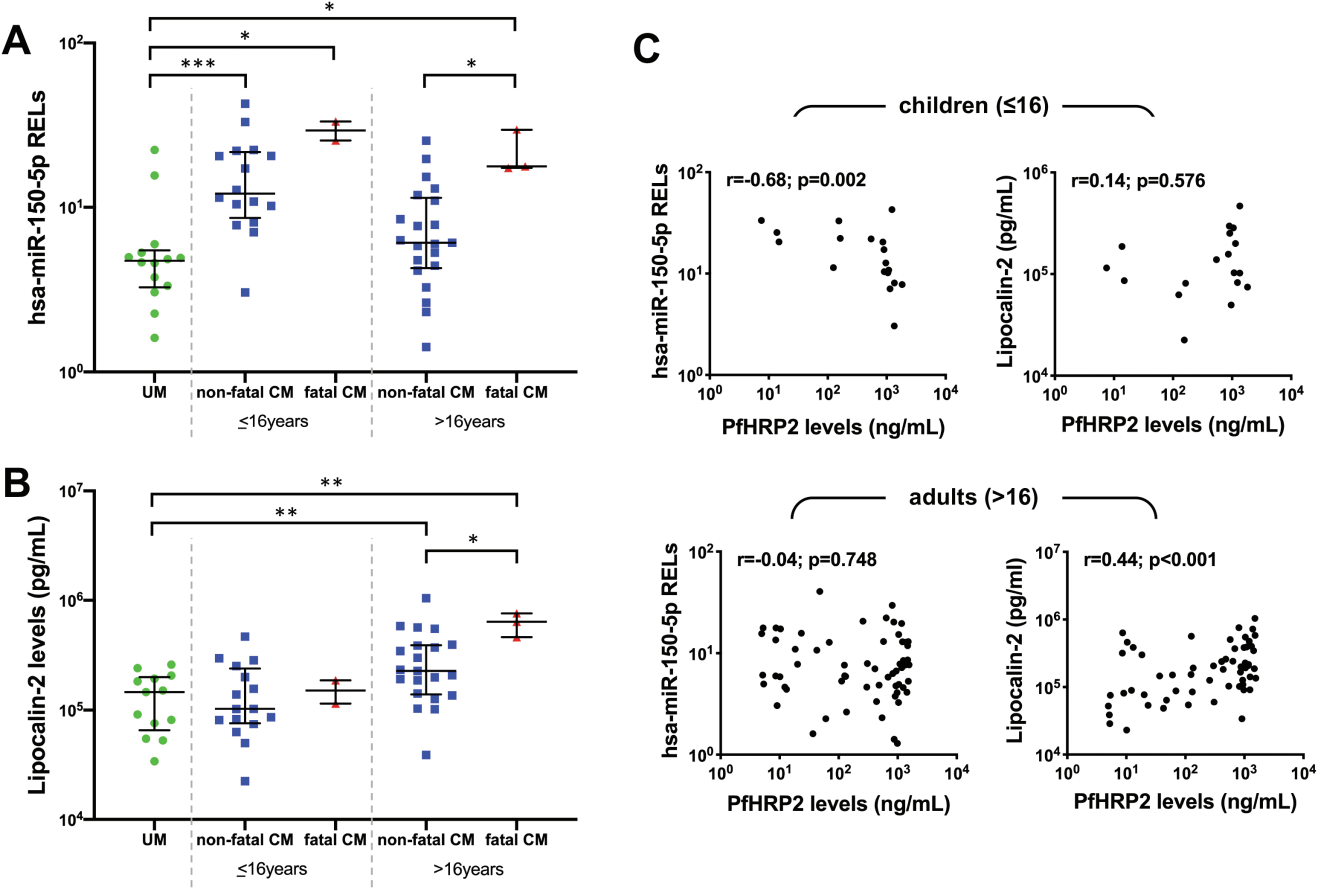
Plasma biomarkers of hypoxia in cerebral malaria (CM). Levels of microRNA 150 (miRNA-150; *A*) and lipocalin-2 (*B*) were measured in the plasma of all patients and plotted according to age and disease category. *C*, Both miRNA-150 and lipocalin-2 were plotted against plasma levels of *Plasmodium falciparum* histidine-rich protein 2 and grouped according to age. Statistical significance was obtained from Mann-Whitney *U* test (*A* and *B*) and Spearman correlation analysis (*C*). *P < .05; **P < .005; ***P < .0005. Abbreviations: CM, cerebral malaria; PfHRP2, *Plasmodium falciparum* histidine-rich protein 2; RELs, relative expression levels; UM, uncomplicated malaria.

Plasma concentrations of miRNA-150 assessed on admission were significantly lower in UM compared to CM patients (median, 4.7 vs 10.6 relative expression levels [RELs], *P* = .002), both with a fatal and nonfatal outcome, and irrespective of age group. The concentration of miRNA-150 on admission discriminated between fatal and nonfatal disease in adults (Figure 4A; median of 25.4 vs 8.5 RELs, *P* = .003), but not in children. Plasma concentrations of lipocalin-2 were not significantly increased in children with CM compared to UM patients, irrespective of the outcome. However, the concentrations of plasma lipocalin-2 were significantly higher in adult patients with CM compared to both pediatric CM and UM cases (median, 266 776 vs 108 797 pg/mL, *P* = .001 and vs 146 062 pg/mL, *P* = .003, respectively). They also discriminated between nonfatal and fatal disease in adult CM (Figure 4B; median, 227 181 vs 636 871 pg/mL, *P* = .0227). Plasma levels of miRNA-150 correlated negatively with PfHRP2 in children (*r* = –0.68, *P* = .002), and lipocalin-2 correlated positively with PfHRP2 in adults (*r* = 0.44, *P* = .001) (Figure 4C).

## DISCUSSION

In this study of both children and adult patients with CM in India, we used quantitative MRI analyses to compare the course of disease between age groups and clinical outcomes. Brain volumes on admission were higher in pediatric compared to adult CM patients and the swelling reversed rapidly in survivors, corroborating previous reports in Malawian children [11]. This age-related difference in brain swelling during acute CM may relate to the more loosely organized extracellular spaces in children, which occupy about 20% of total brain volume and enable a more rapid development of brain swelling compared to adults [21], as described in other diseases [22, 23]. In adult patients with CM, our findings confirm the lack of association between mortality and brain swelling [9, 12].

**Figure 5.**
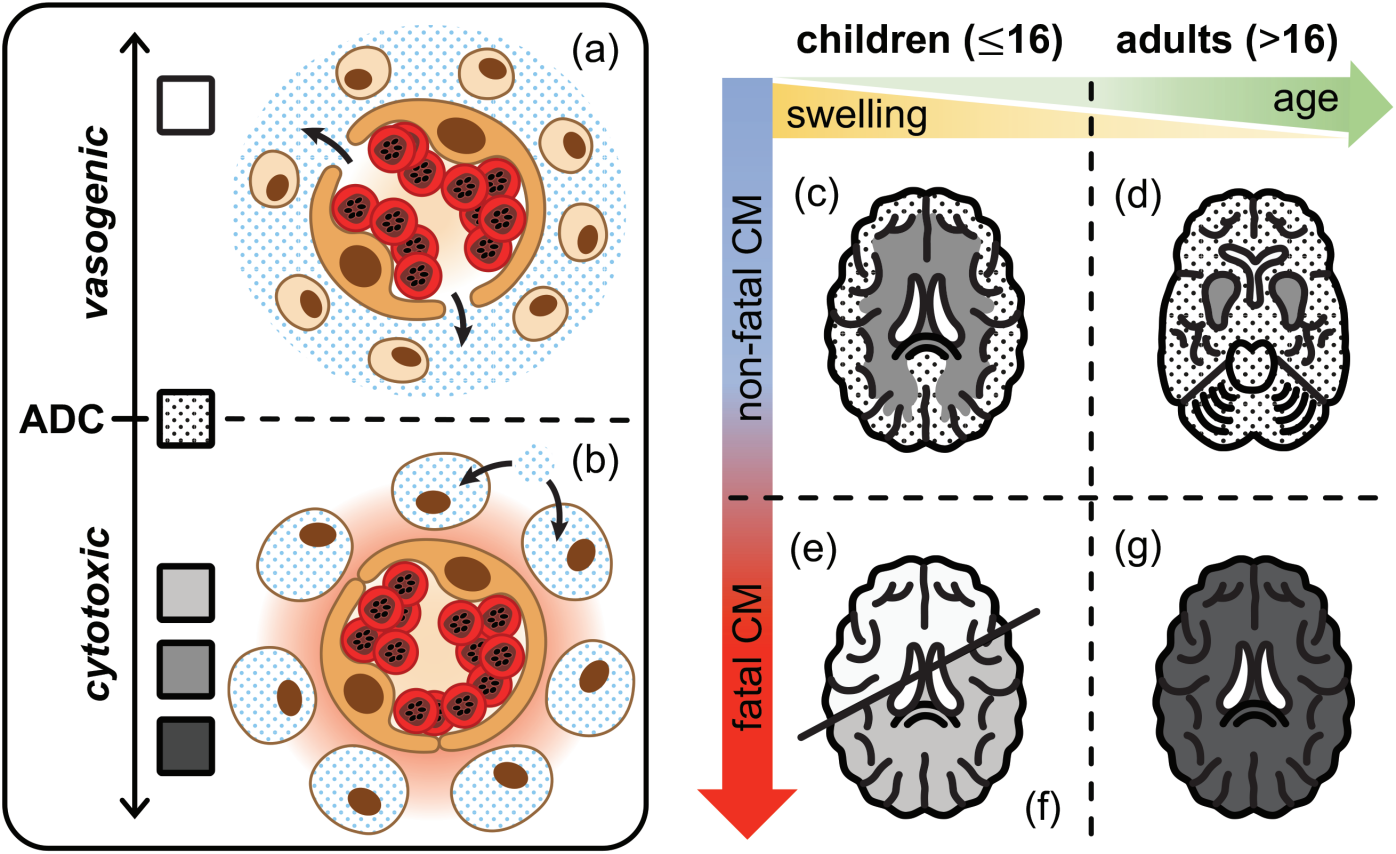
Differences in apparent diffusion coefficient (ADC) values and pathogenic patterns between adults and children with fatal and nonfatal cerebral malaria (CM). An increase in ADC values is associated with extracellular water accumulation in the cerebral tissue. In CM, this is likely to result from vasogenic edema: following blood-brain barrier breakdown, there is a transfer of fluid from the circulation to the brain parenchyma (a). Decreased ADC values are the signature of cytotoxic edema, which is triggered by the obstruction of circulation by sequestered *Plasmodium falciparum*–parasitized red blood cells in CM, resulting in hypoxic and hypoglycemic conditions in the surrounding cerebral tissue (in red). Due to the decreased energy supply, cellular ATP pumps cease to work, causing an osmotic transfer of water inside the cells and their subsequent swelling (b). In nonfatal CM, specific hypoxia-sensitive regions of the brain are affected, and these differ with age: cytotoxic edema evidenced by ADC decrease develops in the white matter in children (c) and in the basal ganglia in adults (d). In both age groups, it reverses rapidly upon antimalaria treatment. In fatal pediatric CM, 2 different patterns were observed: Brain swelling is associated with globally increased ADC signal, indicating diffuse vasogenic edema (e). In contrast, when brain stem herniation occurs, it leads to severe cytotoxic brain swelling with ADC decrease (f). Fatal CM in adult is associated with global, severe hypoxia evidenced by the decreased ADC signal and mild or no brain swelling (g).

In survivors, isolated ADC decrease in hypoxia-sensitive regions was commonly observed in CM patients. Sequestration of pRBCs in the cerebral microvasculature is a hallmark of CM and has long been postulated to alter blood flow, likely resulting in the hypoxic injury we describe [5]. However, these regions differed with age: children predominantly showed restricted diffusion in the white matter, whereas the basal ganglia were mainly affected in adults (Figure 5). The age-related susceptibility of white matter to hypoxia during the acute phase of the pediatric disease may result from active myelination in children, an energy-intensive process sensitive to metabolic disturbances that extends into the third decade of life [24, 25]. Furthermore, the stage of the disease and the degree of hypoxia may contribute to the distinct ADC distribution we identified. Experiments on perinatal primates showed that a milder, more gradual insult resulted in white matter injury sparing the basal ganglia [26, 27], and similar findings were also reported in newborns with neonatal hypoxic-ischemic encephalopathy [28]. In pediatric CM, white matter diffusion restriction may thus reflect prolonged mild/moderate hypoxia. In contrast, a combination of white matter involvement and ADC decrease in the basal ganglia may indicate a more advanced stage of disease [29]. Basal ganglia are areas of high metabolic activity and are highly susceptible to hypoxic changes as they are supplied by end arteries with low collateral blood supply [30]. This may explain the ADC decrease in the basal ganglia of nonfatal adult CM, as similar observations were reported in adults after global hypoxic-ischemic injury [31]. Overall, ADC values in adults were lower upon admission compared to children and increased during recovery, suggesting a stronger cytotoxic component. Plasma levels of miRNA-150, a marker of hypoxia, were significantly higher in CM patients irrespective of the age group, confirming that hypoxia is a frequent occurrence in this neurological syndrome.

In nonfatal disease, ADC values rapidly normalized following treatment with artesunate, indicating reversal of cytotoxic edema, presumably through removal of sequestered pRBCs and restoration of the cerebral microcirculatory blood flow. Some patients showed subtle increased ADC, and this vasogenic component could result from vascular leakage through damaged endothelium after reperfusion [32]. The rapid ADC normalization after treatment and clinical improvement of patients with nonfatal CM within 24–48 hours strongly suggest the involvement of reversible cytotoxic edema [33], and explains why treatment with mannitol aimed at ameliorating vasogenic edema has proven unsuccessful as an adjuvant therapy [9]. In addition, a decrease in blood flow leads to elevated concentrations of the excitotoxic neurotransmitter glutamate [34], causing cell death if glutamate reuptake fails. Glutamine is catalyzed by glutamine synthetase to form glutamate [35]. A recent study showed that treatment with a new glutamine antagonist led to a net decrease of glutamate build-up and prevented mice infected with *Plasmodium berghei* ANKA from developing experimental CM [36], further supporting the role of reversible cytotoxic edema in CM. Remarkably, the UM group in our cohort also showed a subtle whole-brain ADC increase upon admission that reversed after treatment. The slightly elevated ADC values on the first scan suggest mild endothelial dysfunction and vasogenic edema, 2 features that have not previously been reported in fully conscious, nonsevere malaria patients.

We show that fatal CM is associated with global ADC alterations in both age groups, with a more prominent signal decrease in adults. In the absence of brain swelling, this is consistent with a profound global hypoxic injury, likely induced by blood sludging due to sequestered pRBCs [37]. These results contrasted with 2 of 3 pediatric patients who had brain swelling and high ADC values, suggestive of a global accumulation of extracellular fluid. One fatal pediatric case was admitted and scanned at end-stage disease consistent with previous reports [11], with brain stem herniation, consecutive low ADC values due to ceasing blood flow, and resultant cytotoxic edema. Plasma lipocalin-2 levels were significantly higher in adult CM and discriminated between fatal and nonfatal outcomes, confirming severe and global brain hypoxia in fatal adult disease. Lipocalin-2 is released during excitotoxic neuronal injury by neurons and astrocytes [38] and is associated with cerebral hypoxic injury [18]. These previously unreported results demonstrate that profound brain hypoxia measurable by low ADC values is associated with fatality in adult CM, and high plasma levels of miRNA-150 and lipocalin-2 are predictive of negative outcomes. In the adult CM group, plasma levels of lipocalin-2 also correlated positively with PfHRP2, further suggesting that in adults cerebral hypoxia is linked to high parasite burdens and results in more prominent decrease in blood flow compared to children. Although additional studies are warranted in children, this conclusion is in line with the hypothesis that hypoxia may be triggered by a mechanical obstruction of cerebral microvessels by sequestered pRBCs [5], platelets, clumps, and rosettes [39], and immune cells [40].

Our findings suggest for the first time that these distinct disease courses may be differentially targeted by specific adjunctive therapy according to age group. Approaches focusing on a reduction of brain swelling would be more relevant in children and could be achieved by reducing perivascular inflammation. In adults, adjunctive approaches aimed at either ameliorating cytotoxic edema, like glutamate agonists [36], or improving neuroprotection and survival of brain cells may reduce mortality.

## Supplementary Data

Supplementary materials are available at *Clinical Infectious Diseases* online. Consisting of data provided by the authors to benefit the reader, the posted materials are not copyedited and are the sole responsibility of the authors, so questions or comments should be addressed to the corresponding author.

ciaa1647_suppl_Supplementary_Figures_Table

ciaa1647_suppl_Supplementary_Material

## Notes

***Acknowledgments.*** The authors thank the patients and their guardians/families for their participation in this study, as well as Nakul Chandra Khatua and Tapas Kar, the magnetic resonance technicians at Ispat General Hospital, Rourkela, Odisha, India, for their enthusiasm and logistical support. The authors acknowledge Dr Saroj Mishra (deceased) and Professor Frederik Barkhof for their invaluable help during the set-up of the project; the Director in Charge and the clinical staff of Ispat General Hospital in Rourkela for their support and dedication; and the Director of the Institute of Life Sciences in Bhubaneswar for allowing us to use its Infectious Disease Biology Unit to conduct laboratory work in Rourkela, Odisha, India.

***Financial support.*** Research reported in this publication was supported by the National Institute Of Allergy And Infectious Diseases of the National Institutes of Health under Award Numbers U19AI089676 and R21AI142472. The content is solely the responsibility of the authors and does not necessarily represent the official views of the National Institutes of Health. S. C. W. is supported by the Medical Research Council, United Kingdom (award number MR/S009450/1) and A. H. is supported by a clinical leave stipend from the German Centre for Infection Research.

***Potential conflicts of interest.*** The authors: No potential conflicts of interest. All authors have submitted the ICMJE Form for Disclosure of Potential Conflicts of Interest.
