# Supplementary material for: Brain Magnetic Resonance Imaging Reveals Different Courses of Disease in Pediatric and Adult Cerebral Malaria
Source: Clin Infect Dis. 2020 Dec 16;73(7):e2387–96. doi: 10.1093/cid/ciaa1647 (PMC8492227; doi:10.1093/cid/ciaa1647)
Supplement: ciaa1647_suppl_Supplementary_Figures_Table [file ciaa1647_suppl_supplementary_figures_table.pdf]

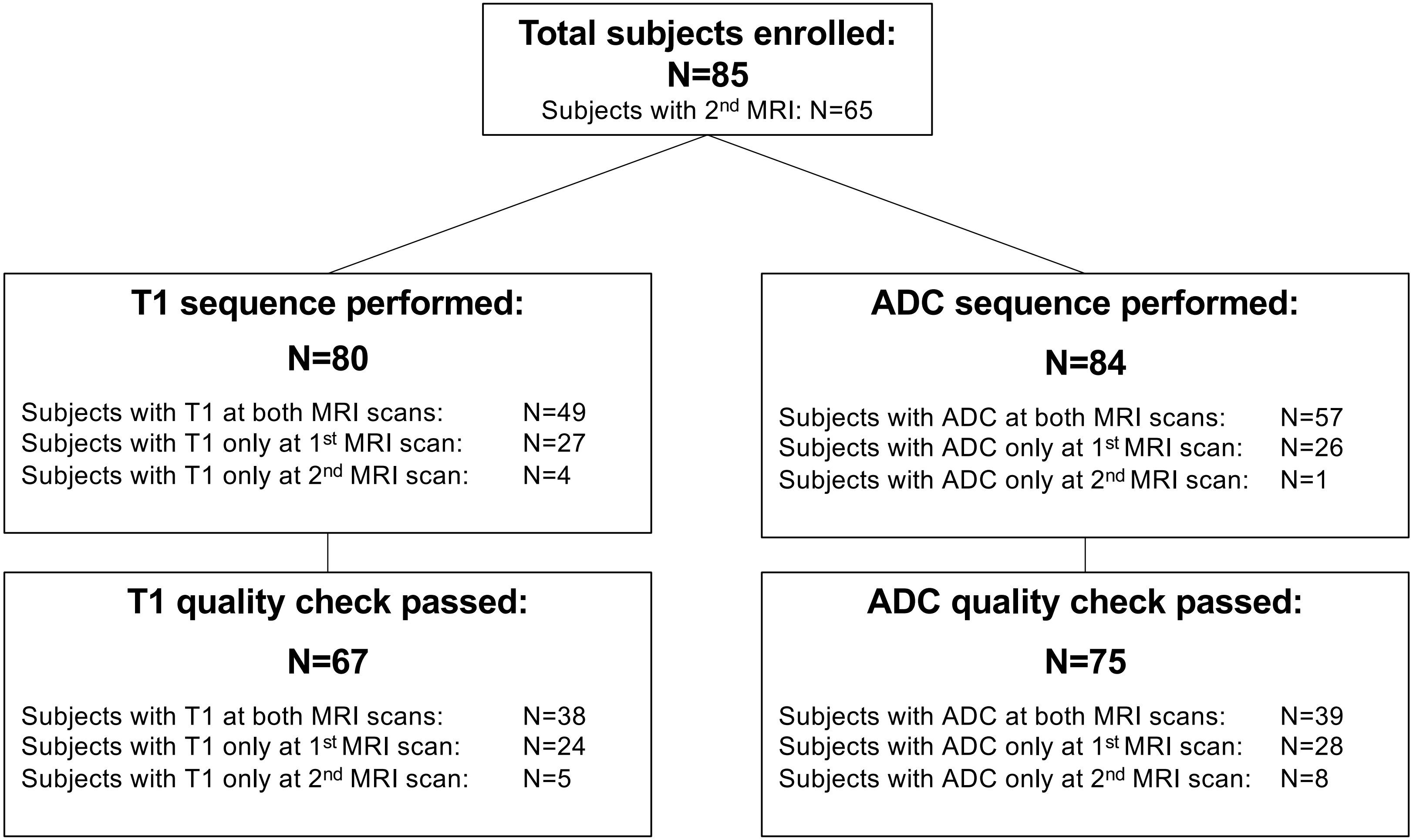
Total subjects enrolled:
N=85
Subjects with 2nd MRI: N=65
T1 sequence performed:
N=80
Subjects with T1 at both MRI scans: N=49
Subjects with T1 only at 1st MRI scan: N=27
Subjects with T1 only at 2nd MRI scan: N=4
ADC sequence performed:
N=84
Subjects with ADC at both MRI scans: N=57
Subjects with ADC only at 1st MRI scan: N=26
Subjects with ADC only at 2nd MRI scan: N=1
T1 quality check passed:
N=67
Subjects with T1 at both MRI scans: N=38
Subjects with T1 only at 1st MRI scan: N=24
Subjects with T1 only at 2nd MRI scan: N=5
ADC quality check passed:
N=75
Subjects with ADC at both MRI scans: N=39
Subjects with ADC only at 1st MRI scan: N=28
Subjects with ADC only at 2nd MRI scan: N=8

S1

Step:	Programme	Input image	Reference image	Degrees of freedom	Additional options	Algorithm
**1. ALIGNMENT** (brain extracted T1)	FSL-FLIRT (linear registration)	MNI152 T1-2mm brain	Brain extracted native T1	6	search-range ±45°	mutual information
**2. SKULL SCALING**	FSL-FLIRT (linear registration)	MNI152 T1-2mm	Native T1	12	nosearch, initialization with registration matrix of step 1	mutual information
**3. SKULL REMODELING**	FSL-FNIRT (non-linear registration)	MNI152 T1-2mm	Native T1	NA	initialization with registration matrix of skull-scaling step 2	NA
**4. TRANSFORMATION APPLICATION** (on skull mask)	FSL-applywarp	Skull mask in MNI152 space	Native T1	NA		NA
**5. SCALING FACTOR**	FSL-fslstats	Registrated skull mask in native T1 space, thresholded at 0.5	Skull mask in MNI152 space	NA	NA	NA

FSL-FLRIT, FSL-FNIRT, FSL-bet and FSL-applywarp are part of the FSL FMRIB Software Library v6.0, freely available at: https://fsl.fmrib.ox.ac.uk/fsl/

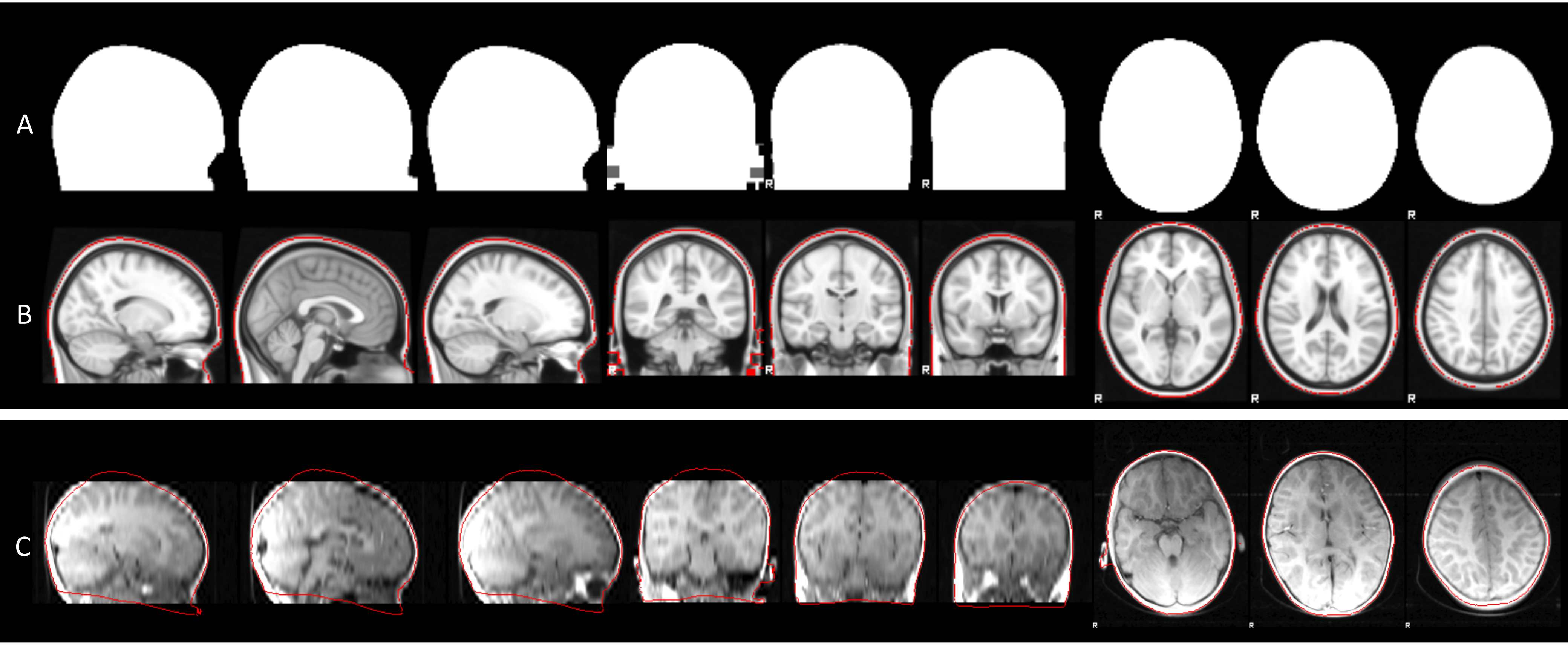
A
B
C

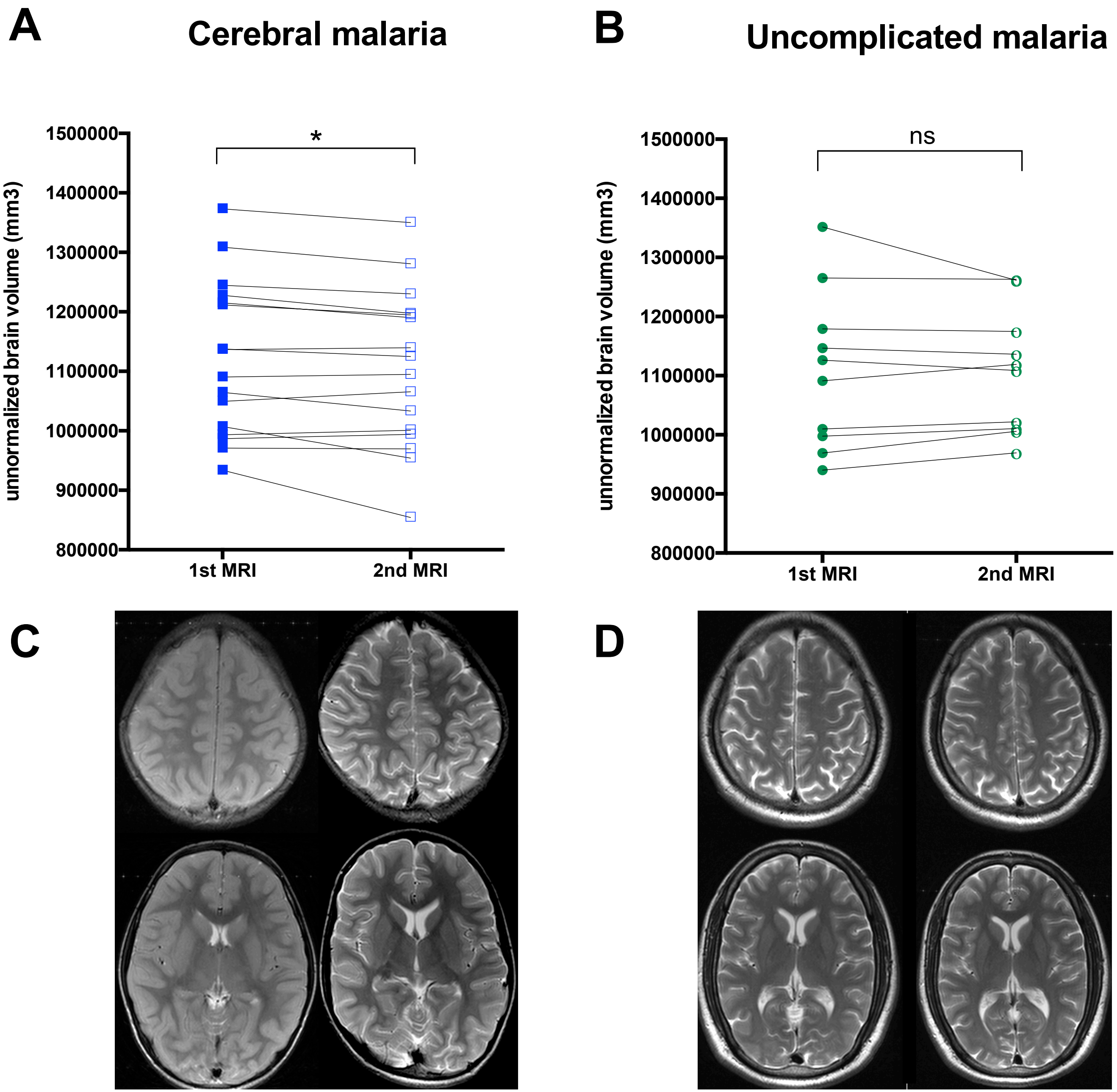
A
Cerebral malaria
B
Uncomplicated malaria
unnormalized brain volume (mm3)
1500000
1400000
1300000
1200000
1100000
1000000
900000
800000
*
ns
1st MRI
2nd MRI
C
D

S4

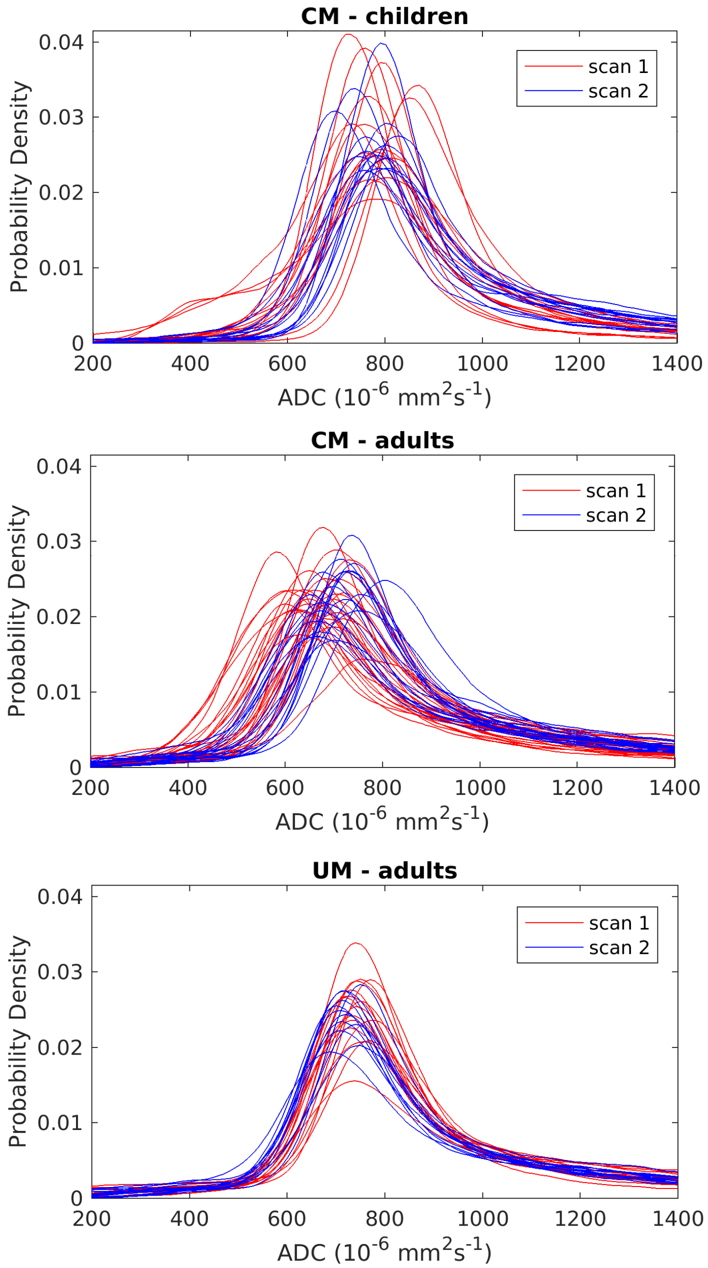
CM - children
Probability Density
0.04
0.03
0.02
0.01
0
200
400
600
800
1000
1200
1400
ADC (10-6 mm2s-1)
scan 1
scan 2
CM - adults
Probability Density
0.04
0.03
0.02
0.01
0
200
400
600
800
1000
1200
1400
ADC (10-6 mm2s-1)
scan 1
scan 2
UM - adults
Probability Density
0.04
0.03
0.02
0.01
0
200
400
600
800
1000
1200
1400
ADC (10-6 mm2s-1)
scan 1
scan 2

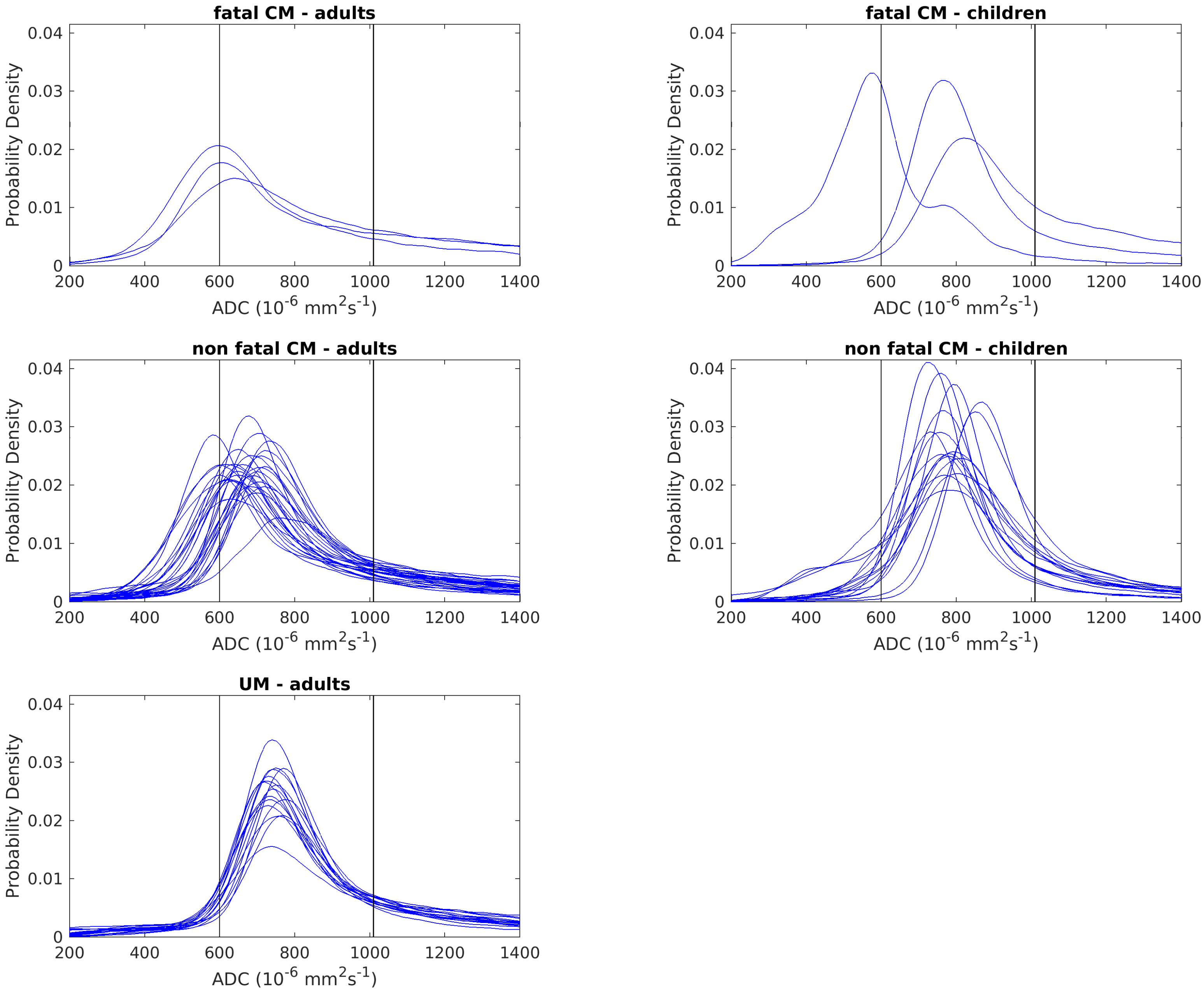
fatal CM - adults
fatal CM - children
non fatal CM - adults
non fatal CM - children
UM - adults
Probability Density
0 0.01 0.02 0.03 0.04
200 400 600 800 1000 1200 1400
ADC (10^{-6} mm^2s^{-1})

S6

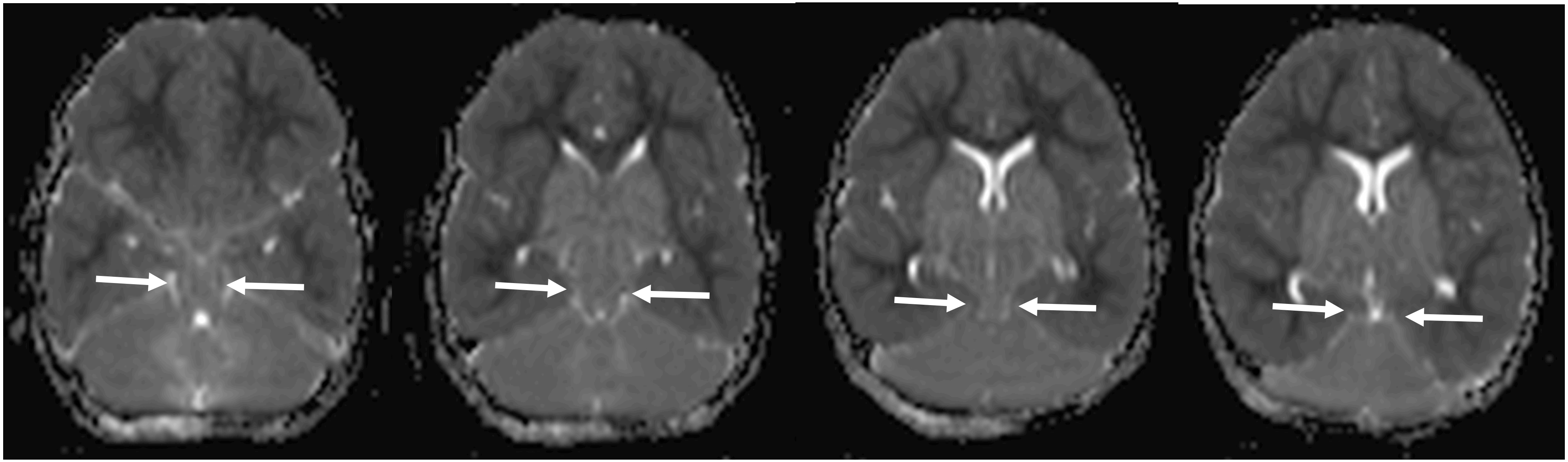

S7
