# Supplementary material for: Brain Magnetic Resonance Imaging Reveals Different Courses of Disease in Pediatric and Adult Cerebral Malaria
Source: Clin Infect Dis. 2020 Dec 16;73(7):e2387–96. doi: 10.1093/cid/ciaa1647 (PMC8492227; doi:10.1093/cid/ciaa1647)
Supplement: ciaa1647_suppl_Supplementary_Material [file ciaa1647_suppl_supplementary_material.docx]

**SUPPLEMENTARY MATERIAL**

1. **PATIENTS AND METHODS**
2. *Inclusion and exclusion criteria for CM patients*

Consecutive patients with coma persisting after correction of hypoglycemia (<2.2 mmol/L), and a proven *Plasmodium falciparum* infection (detected by rapid diagnostic test and confirmed by the presence of asexual forms of the parasite in a peripheral blood smear) were eligible for inclusion. Patients who were clinically unstable because of hemodynamic shock (systolic blood pressure <80 mmHg with cool extremities), or signs of respiratory insufficiency (respiratory rate above 30/min, nailbed oxygen saturation <90% by pulse oximetry) were excluded because of the increased risk incurred by being transported to the MRI facilities. Additional exclusion criteria are detailed elsewhere [1]. Due to the lack of a direct association between the presence of retinopathies and CM in low malaria transmission settings, retinal changes were documented but not used as a diagnostic parameter. Tonic-clonic seizures were diagnosed based on clinical observation. An age cutoff of 16 was used to separate children from adults in our cohort, based on previously published results demonstrating that the peak of total brain volume is reached at 15.2 years [2].

1. *Clinical care*

All CM patients were treated with intravenous artesunate (2.4 mg/kg of body weight STAT, 12 and 24 hours, and then daily until able to take an oral artemisinin-based combination therapy, and with a minimum of three intravenous doses. Patients with UM were treated with oral artesunate (4mg/kg of body weight) once daily for three days together with a single dose of oral sulfadoxine/pyrimethamine (25 mg/kg and 1.25 mg/kg of body weight) on the first day of oral therapy. A single gametocytocidal dose of primaquine (0.75 mg/kg of body weight) was given on the second day of oral therapy.

1. *Retinal examination*

To compare potential differences in pathogenetic processes between retinal and cerebral vasculatures in our cohort of CM cases [3], all patients underwent retinal examination by direct and indirect ophthalmoscopy within 6 hours of admission, and the severity of the findings was graded by two ophthalmologists (RRM, SJ, Table 1), according to published classification criteria [4].

1. *MR imaging sequence and dataset exclusions*

The MRI sequence protocol included: axial T2-weighted (turbo spin echo, TE=99ms/TR=4000ms, FA=150°, resolution=0.9x0.9x5mm), T1-weighted (spin echo, TE=7.7ms/TR=500ms, FA=90°, resolution=0.9x0.9x5mm), fluid-attenuated inversion recovery (FLAIR) (TE=114ms/TR=9000ms, TI=2500ms, FA=150°, resolution=0.9x0.9x5mm), diffusion weighted imaging (DWI) (EPI, TE=108ms/TR=3500ms, FA=90°, b-values 0, 500, 1000 s/mm^2^, resolution=1.8x1.8x5mm) and sagittal T2-weighted images (turbo spin echo, TE=99ms/TR=3800ms, FA=150°, resolution=0.9x0.9x5mm). One non-fatal CM patient with clinically reported seizures showed a typical seizure-induced imaging pattern with only cortical diffusion restriction [5] and was excluded from the analysis. Due to motion artefacts or image distortions (gradient nonlinearities), 22 ADC maps (13 on admission, nine follow-up) were excluded.

1. *SIENAX and SIENA and dataset exclusions*

SIENA X was used to assess brain volume, and SIENA to compare follow-up scans [6]. Both programs are part of the FSL toolbox (<https://fsl.fmrib.ox.ac.uk/fsl>). Due to the large variation of pediatric skull shape compared to adults, normalized brain volume was assessed by nonlinear registration of the skull into the MNI standard space using FSL-FLIRT and FSL-FNIRT (see Supplementary Fig. 2 and 3) [7]. Additional visual quality checks prompted the exclusion of 12 datasets (eight on admission, four follow-ups; 11 non-fatal, one fatal CM)

1. *Normalized ADC histograms*

Normalized ADC histograms were created with a bin-size of 10 mm^2^s^-1^$\times$10^6^, a range between 0 and 2500 mm^2^s^‑1^$\times$10^6^ and normalized to the total number of voxels [8], using an in-house developed MATLAB script. Histograms were smoothed using the MATLAB standard smoothing-spline fitting algorithm.

1. *Evaluation of plasma levels of Lipocalin-2, microRNA-150, and PfHRP2*

Whole blood specimens (4.5 mL) were collected in sodium citrate tubes upon admission for all patients. Specimens were then immediately centrifuged at 500 *x g* for 10 minutes and plasma aliquots stored at -80°C prior to batch analyses. Lipocalin-2 was quantified using a multiplexed bead-based enzyme-linked immunosorbent assay (Luminex Corp, Austin, USA) via a commercially available customizable assay kit (Human Magnetic Luminex Assay, R&D Systems, Minneapolis, USA). hsa-miR-150-5p was measured after 50µl of plasma per patient was subjected for RNA extraction using the miRNeasy Plasma/Serum kit (Qiagen), with the addition of 5µg UltraPure™ glycogen per sample (Invitrogen). A synthetic RNA oligo version of cel-miR-39-3p was included, following lysis reaction as per the manufacturer’s instructions. cDNA synthesis and RT-qPCR with an ABI 7500 Fast Real-Time PCR System (Applied Biosystems, Foster City, USA) were performed using TaqMan® Advanced miRNA assays. cDNAs were selected randomly to prepare serially diluted five points standard curve and was run in triplicate for each miRNA. Data generated by RT-qPCR was normalized using a mean Ct value of two endogenous controls (ECs, hsa-miR-30d-5p and hsa-miR-191-5p) [9]. Relative expression levels (RELs) of hsa-miR-150-5p were calculated with the ∆Ct method [Ct (miRNA) – Mean Ct (ECs)], considering efficiencies of 100% for all the miRNAs and ECs [10]. Quality control of the miRNA measurements showed that samples amplified for exogenous control (cel-miR-39-3p) had a Ct value <24 and a coefficient of variance <5%, which indicates a correct cDNA preparation. PCR efficiencies for target miRNA (hsa-miR-150-5p), exogenous (cel-miR-39-3p) and endogenous (hsa-miR-30d-5p and hsa-miR-191-5p) controls were in the range of 98-106%. PfHRP2 was quantified by commercially available ELISA kits (CellLabs, Sydney, Australia).

**REFERENCES**

1. Mohanty S, Benjamin LA, Majhi M, et al. Magnetic Resonance Imaging of Cerebral Malaria Patients Reveals Distinct Pathogenetic Processes in Different Parts of the Brain. mSphere **2017**; 2(3).

2. Narvacan K, Treit S, Camicioli R, Martin W, Beaulieu C. Evolution of deep gray matter volume across the human lifespan. Hum Brain Mapp **2017**; 38(8): 3771-90.

3. Greiner J, Dorovini-Zis K, Taylor TE, et al. Correlation of hemorrhage, axonal damage, and blood-tissue barrier disruption in brain and retina of Malawian children with fatal cerebral malaria. Front Cell Infect Microbiol **2015**; 5: 18.

4. Harding SP, Lewallen S, Beare NA, Smith A, Taylor TE, Molyneux ME. Classifying and grading retinal signs in severe malaria. Trop Doct **2006**; 36 Suppl 1: 1-13.

5. Kim SE, Lee BI, Shin KJ, et al. Characteristics of seizure-induced signal changes on MRI in patients with first seizures. Seizure **2017**; 48: 62-8.

6. Smith SM, De Stefano N, Jenkinson M, Matthews PM. Normalized accurate measurement of longitudinal brain change. J Comput Assist Tomogr **2001**; 25(3): 466-75.

7. Jenkinson M, Bannister P, Brady M, Smith S. Improved optimization for the robust and accurate linear registration and motion correction of brain images. Neuroimage **2002**; 17(2): 825-41.

8. Tofts PS, Davies GR, Dehmeshki J. Histograms: Measuring Subtle Diffuse Disease. In: Quantitative MRI of the Brain: Measuring Changes Caused by Disease. In: Tofts P: John Wiley & Sons, Ltd, **2003**.

9. Gupta H, Rubio M, Sitoe A, et al. Plasma microRNA profiling for malaria disease: association with severity and P. falciparum biomass. MedRxiv **August 4, 2020**; Available from: <https://www.medrxiv.org/content/10.1101/2020.07.31.20165712v1>.

10. Marabita F, de Candia P, Torri A, Tegner J, Abrignani S, Rossi RL. Normalization of circulating microRNA expression data obtained by quantitative real-time RT-PCR. Brief Bioinform **2016**; 17(2): 204-12.

1. **SUPPLEMENTARY TABLE**

**Supplementary Table 1: Admission and enrolment figures at IGH for the period of the study**

| Year | Total febrile patients | Malaria-positive patients | *P. falciparum-*positive patients | Study eligible patients (ALL) | Study eligible patients (CM) | Screen failures (ALL)* | Screen failures (CM)* | Recruited patients (ALL) | Recruited patients (CM) |
| --- | --- | --- | --- | --- | --- | --- | --- | --- | --- |
| 2013 | 693 | 113 | 102 | 23 | 10 | 13 | 4 | 10 | **6** |
| 2014 | 2,119 | 235 | 199 | 16 | 10 | 03 | 3 | 13 | **7** |
| 2015 | 1,542 | 176 | 144 | 70 | 37 | 12 | 11 | 58 | **26** |
| 2016 | 3,328 | 326 | 304 | 32 | 26 | 6 | 6 | 26 | **20** |
| 2017 | 2,926 | 186 | 170 | 9 | 6 | 0 | 0 | 9 | **6** |
| 2018 | 1,971 | 44 | 34 | 10 | 2 | 0 | 0 | 10 | **2** |
| 2019 | 1,613 | 28 | 27 | 11 | 4 | 2 | 2 | 9 | **2** |
| **TOTAL** | **14,192** | **1,108** | **980** | **171** | **95** | **36** | **26** | **135** | **69**** |

ALL: total of CM, UM and severe, non-CM patients

*due to enrolment criteria not being met (see section I.1 above), family or patient declining to participate, or other reasons.

**4 enrolled CM patients were excluded due to severe motion artefacts during the scanning, leading to a total of 65 CM patients included in the study

1. **SUPPLEMENTARY FIGURE LEGENDS**

**Supplementary Figure 1: Consort diagram of the study.** A total of 85 patients were enrolled and scanned, and 65 individuals underwent a second scan. T1 and ADC sequences were performed in 80 and 84 individuals, respectively. Quality controls were performed for both sequences and passed for 67 (T1) and 75 (ADC) patients.

**Supplementary Figure 2: Registration scheme for the optimized brain volume normalization.** The brain volume normalization of SIENAX calculates a scaling factor by using a linear registration to register the skull of a subject into the skull of a standard template (MNI-152) (see: https://fsl.fmrib.ox.ac.uk/fsl/fslwiki/SIENA). However, linear transformations do not account for different skull shapes, which is problematic as these may differ between patients: this issue was observed in pediatric cases from our cohort. Therefore, we used an additional nonlinear registration step to calculate the scaling factor for each subject, as described in the registration scheme. In **step 1**, the brain extracted T1 image of the standard space (MNI152-T1_2mm.brain) was aligned to the brain extracted T1 image of each subject by preserving the brain size (rigid registration). The brain extraction was performed with FSL-bet. In **step 2**, the size of the aligned standard-skull was scaled to the skull of the subject. In order to properly match both skulls and to account for different skull shapes, skull remodeling (**step 3**) was performed with a non-linear registration using FSL-FNIRT, obtaining a nonlinear registration file (warp-file) for each subject. In **step 4**, the skull mask of the standard template (Fig. S2 A) was transformed into the native T1 space of each subject using the obtained warp-files (shown for one representative patient in Fig. S2 C). In the final step, the volumes of the standard space skull and the subject-skull masks were measured with fslstats and the scaling factor was calculated by taking the ratio over both volumes.

**Supplementary Figure 3: Optimized brain volume normalization.** Binary skull mask in the standard space (MNI152) (A), and corresponding T1 image with the outlines of the skull masks are overlaid in red (B). Native T1 scan of a representative patient with the outline of the transformed skull mask resulting from the described registration scheme, is overlaid (C). By comparing the overlaid skull outline with the bright skull voxels of the underlying T1 image, the quality of the registration process could be determined for each individual.

**Supplementary Figure 4: Evolution of brain volume between admission and follow-up scans:** Variation of unnormalized brain volume for each individual in non-fatal CM (A) and UM (B) patients. Brain swelling in CM patients had normalized on the second MRI scan (A), UM did not show significant differences of brain volume between admission and follow-up except for one patient (B). Representative images at two levels on axial T2-weighted images show that reduced cerebrospinal fluid spaces on admission reverse at follow-up in non-fatal CM (C). No volume differences were noted by visual inspection between the two scans in UM patients (D).

**Supplementary Figure 5: Evolution of ADC histograms between admission and follow-up.** ADC histograms on admission (red) and at follow up (blue) from all subjects are displayed and grouped according to brain region and age.

**Supplementary Figure 6: Comparison of whole-brain ADC histograms on admission between age and disease groups.** Histograms generated for all patients are displayed and grouped according to age and outcome. Note: vertical black bars are intended as a visual support to facilitate comparisons between groups. They were calculated from the average of all adults UM histograms and overlaid on all graphs. The left bar represents the lower ADC value at 25% of histogram maximum, the right bar is derived from the corresponding high ADC value of the 25% of histogram maximum.

**Supplementary Figure 7: ADC maps of the fatal pediatric case with end stage disease**. Signs of downward transtentorial brain herniation are clearly visible with no more fluid spaces in the basal cistern (white arrows). Supratentorial swelling is extensive with ADC decrease and no more adjacent fluid spaces.
